# Genome‐wide associations of leaf spectral variation in MAGIC lines of *Nicotiana attenuata*

**DOI:** 10.1002/ecy.70366

**Published:** 2026-04-12

**Authors:** Cheng Li, Ewa A. Czyż, Bernhard Schmid, Rishav Ray, Rayko Halitschke, Ian T. Baldwin, Michael E. Schaepman, Meredith C. Schuman

**Affiliations:** ^1^ Department of Geography University of Zürich Zürich Switzerland; ^2^ Department of Molecular Ecology Max Planck Institute for Chemical Ecology Jena Germany; ^3^ Department of Chemistry University of Zürich Zürich Switzerland; ^4^ Present address: Jet Propulsion Laboratory California Institute of Technology Pasadena California USA; ^5^ Present address: Department of Plant Biology University of California Davis California USA

**Keywords:** field spectroscopy, genome‐wide association studies (GWAS), hierarchical spectral clustering with parallel analysis, leaf reflectance, multiparent advanced generation inter‐cross (MAGIC), *Nicotiana attenuata*

## Abstract

The application of in‐field and aerial spectroscopy to assess functional and phylogenetic variation in plants has led to novel ecological insights and supports global assessments of plant biodiversity. Understanding how plant genetic variation influences reflectance spectra will help harness this potential for biodiversity monitoring and improve understanding of why plants differ in functional responses to environmental change. Here, we use a well‐resolved genetic mapping population derived from Multiparent Advanced Generation Inter‐cross (MAGIC) lines of *Nicotiana attenuata* to associate genetic differences with differences in leaf spectra between plants in a field experiment in their natural environment. We analyzed the leaf reflectance spectra using a hand‐held spectroradiometer (350–2500 nm) on 616 fully genotyped plants of *N. attenuata* grown in a randomized block design. We tested three approaches to conducting genome‐wide association studies on spectral variants. We introduce a new hierarchical spectral clustering with parallel analysis (HSC‐PA) method. This method efficiently captured the variation in our high‐dimensional dataset and allowed us to discover a novel association, between a locus on chromosome 1 and the 734–1143 nm spectral range, spanning the red‐edge and near‐infrared regions that are sensitive to leaf structure and photosynthetic activity. This locus contains a candidate gene annotated as carbonic anhydrase, an enzyme involved in CO₂ hydration and regulation of photosynthetic efficiency, suggesting a physiological link between variation in leaf optical properties and carbon assimilation. In contrast, an approach treating single wavelengths as phenotypes identified genetic signals highly consistent with HSC‐PA, but suffered from massive statistical redundancy without pinpointing significant, interpretable features. An index‐based approach, which reduces complex spectra to a few dimensionless variables, detected two significant associations for $\text{ARDSI}\_C_{w}$ (a water‐content‐related index) with loci on chromosome 1 near genes annotated as a Zeta toxin domain‐containing protein, and an Exocyst subunit Exo70 family protein. While these findings are biologically plausible, they represent a very narrow subset of the spectral variation captured by HSC‐PA. The HSC‐PA approach supports a comprehensive understanding of the genetic determinants of leaf spectral variation that is data‐driven but human‐interpretable and is thus a tool to discover genetic differences underlying intraspecific variation, a foundation of biodiversity.

## INTRODUCTION

Genetic diversity is essential for the survival and adaptation of species to changing environmental conditions. Radiation reflected, absorbed, and transmitted by plants constitutes the basis for remote sensing of vegetation, commonly in the range from visible (VIS) to infrared wavelengths corresponding to solar radiation (Knipling, 1970; Thenkabail et al., 2018). Remote sensing may revolutionize the way we study genetic diversity by offering noninvasive methods with the potential of repeated measurement on large spatial scales (Madritch et al., 2014). Remote sensing technologies have not only facilitated the study of Earth's surface features, including vegetation, water bodies, and land‐use patterns, but are also gaining applications in biodiversity research. Notably, high‐throughput hyperspectral phenotyping has long been used in crop quantitative genetics to link genetic variation with spectral traits, demonstrating the heritability of multiple biochemical and physiological traits estimated from spectral signatures—thus providing valuable methodological precedents for biodiversity and ecological studies (Grzybowski et al., 2021). Wang and Gamon (2019) emphasized the potential of “optical diversity”—spectral variation driven by underlying chemical and structural traits—as a scalable proxy for monitoring plant biodiversity. For example, Asner and colleagues showcased the capabilities of high‐fidelity imaging spectroscopy for mapping the diversity of tropical forest canopies (Asner et al., 2014). Although they do not capture the structural variation of whole canopies, reflectance spectra from single leaves, which can be captured with controlled lighting and background from plants in field conditions, also offer insights into plant physiology, stress responses, and genetic variation, with recent studies establishing a correlation between genetic diversity and leaf reflectance spectra (Asner et al., 2015; Cavender‐Bares et al., 2016; Czyż et al., 2023; Serbin et al., 2014; Stasinski et al., 2021; Wang et al., 2018). We recently showed that genetically variable plant populations are also spectrally more variable in comparison with replicates of an inbred genotype grown under either glasshouse or field conditions, while isogenic plants differing primarily in their gene expression are spectrally similar to replicates of the inbred genotype from which they were derived (Li, Czyz, et al., 2023; Li, Czyż, et al., 2023).

A genome‐wide association study (GWAS) is a powerful tool used to identify genetic variants associated with specific traits or diseases in populations. By examining the entire genome, researchers can pinpoint specific genetic markers that correlate with phenotypic variation. A primary advantage of GWAS is that it requires no prior knowledge of potential candidate genes and can be used for the discovery of novel genetic associations (Santure & Garant, 2018; Visscher et al., 2017). The corresponding disadvantage of GWAS is that it does not provide causal links and thus the mechanisms underlying statistical associations must be dissected and tested. A primary concern of GWAS is the influence of population structure, which, if not addressed, may produce misleading associations (Price et al., 2006). Population structure refers to the presence of subgroups within a population, which differ in allele frequencies due to shared ancestry. In GWAS, population structure can be confounding because genetic variants associated with the subgroup, rather than the trait of interest, may result in false‐positive associations. To mitigate this, various methods, such as principal components analysis (PCA) and mixed linear models (Zhang et al., 2010), are employed to correct for population structure in GWAS analyses.

However, the power of these genomic analyses relies heavily on the availability of precise, large‐scale trait data. Phenotyping remains a substantial bottleneck in genetic studies due to its labor‐intensive and time‐consuming nature. High‐throughput phenotyping, especially hyperspectral imaging, offers a powerful solution—enabling rapid, scalable, and noninvasive measurements of plant traits that can accelerate genotype–phenotype association studies (Furbank & Tester, 2011; Zhang & Zhang, 2018).

*Nicotiana attenuata* Torr. ex S. Watson is a model wild plant for molecular ecology native to the Great Basin Desert of the southwestern United States. It is primarily found in large ephemeral populations following fires in sagebrush and pinyon–juniper ecosystems. Its unique germination behavior is stimulated by cues found in wood smoke and the removal of inhibitors from unburned litter, allowing it to thrive in postfire environments (Bahulikar et al., 2004). Ecologically, *N. attenuata* is a compelling subject for its intricate defense mechanisms against herbivores, its tissue‐specific diurnal metabolic rhythms, and its ability to acclimate to varying environmental conditions, including high UVB radiation (Ðinh et al., 2013; Glawe et al., 2003; Kim et al., 2011; Li et al., 2016). Furthermore, it offers unique opportunities for genetic research due to a well‐defined Multiparent Advanced Generation Inter‐Cross (MAGIC) population (Ray et al., 2019, 2023). This design comprises 26 phenotypically and genetically differentiated parental lines (PLs) intercrossed to produce two replicate sets each of 325 recombinant inbred lines (RILs; 616 genotyped plants of the total of 650 used in the present study), allowing for the study of phenotype–genotype associations without the confounding influence of population structure. More details on plant material, field site, and generation of MAGIC RIL population can be found in Appendix S1: Section S1.

**FIGURE 1:**
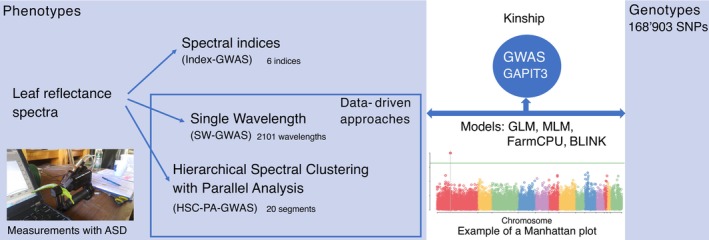
Approaches and toolbox of this study. Photo credit: Meredith C. Schuman. ASD, analytical spectral devices; BLINK, Bayesian information and linkage‐disequilibrium iteratively nested keyway; FarmCPU, fixed and random model circulating probability unification; GLM, general linear model; GWAS, genome‐wide association study; MLM, mixed linear model; SNP, single nucleotide polymorphism; SWs, single wavelength.

In this study, we explore the genetic diversity of the MAGIC population to identify associations between genetic variants and leaf spectral traits, advancing our understanding of genetic influences on complex spectral characteristics. Our methodology involves three approaches: analyzing spectral indices, single wavelengths (SWs), and implementing hierarchical spectral clustering with parallel analysis (HSC‐PA). Through GWAS using 168,903 single nucleotide polymorphisms (SNPs) that showed variation within the population, we investigate genetic links with genome annotation. Our methodological approach and the tools utilized are summarized in Figure 1.

## MATERIALS AND METHODS

This study's methodology extends from our previous work (Li, Czyz, et al., 2023; Li, Czyż, et al., 2023), with detailed description available in Appendix S1: Section S1. Briefly, our study utilizes two replicate sets each of 325 *N. attenuata* MAGIC RILs, derived from 26 PLs and 6 generations of inbreeding, yielding a total of 650 genetically different plants, of which 616 were planted in the current experiment. The RIL sequencing was performed at Novogene HK (Ray et al., 2023). Leaf optical properties (350–2500 nm) were measured using a FieldSpec 4 spectroradiometer (ASD Inc., Boulder, CO, USA). Key procedures are summarized here, with technical specifications provided in Appendix S1: Section S1 and full protocols in Li, Czyz, et al. (2023) and Li, Czyż, et al. (2023).

Measurements were conducted on batches of hydrated, freshly cut leaves to allow high‐throughput and standardized measurement conditions. For each sample, raw radiance scans were collected under four conditions (white and black references, with and without leaf). Data processing was performed using R (R Core Team, 2023), employing the spectrolab package (version 0.0.10, Meireles et al., 2017). Reflectance values from a calibrated instrument, normalized to the same white background that was used for leaf measurements, were used to calculate relative reflectance with a formula derived from Miller et al. (1992), which accounts for both background reflectance and instrument noise. We restricted the analysis to the 400–2500 nm spectral range, excluding the 350–399 nm region due to especially low signal‐to‐noise ratios. Prior to statistical modeling, we applied a strict three‐step outlier removal procedure (visual inspection, local outlier factor analysis, and final verification) to generate the filtered spectral dataset.

We then employed a linear model to remove known environmental effects from the raw spectral data. Specifically, for each wavelength, we fitted the model:

$$Y\sim \mathrm{LN}+\mathrm{REP}+\mathrm{BA}+\mathrm{LN}:\left(\mathrm{REP}+\mathrm{BA}\right)+\mathrm{DT}:\mathrm{CT}+\mathrm{MO}$$

The model terms are defined as follows:

1. LN (leaf number): Represents the influence of the measurement technique (the number of measured leaves used to fill the leaf clip device: one or two).
2. REP (replicate): Accounts for potential differences between the two sets of RILs (that occupied different sectors of the field plot and were measured in series).
3. BA (patch): Represents spatial patch effects characterized from *N. attenuata* reference measurements (in each square of 2 × 2 planting positions, one was occupied by a “phytometer” plant of a single tester genotype of the species).
4. $\mathrm{LN}:\left(\mathrm{REP}+\mathrm{BA}\right)$: Models the interaction between measurement technique (LN) and spatial effects (REP, BA).
5. DT:CT: captures temporal effects (main effects of day, DT, and time of day, CT, and their interaction).
6. MO (maternal origin): Denotes maternal cytoplasmic effects.

The residuals from each fitted model were taken as the adjusted spectral values, corrected for (i.e., removing) the modeled environmental influences. This approach minimizes systematic environmental noise to improve the detection of heritable spectral traits. Alternatively, environmental effects could have been removed in the mixed models of the GWAS analysis described below, but this would have been more complicated, in particular with regard to the correction for the patch effects using the phytometer plants (BA). Note that effects of genotype‐by‐environment interactions cannot be removed, because this would also remove main effects of genotypes, which must be retained for the subsequent GWAS analysis.

We tested three ways of treating the spectral phenotypic data. First, we used six selected spectral indices, which are single values derived from ratios of reflectance at specific wavelengths representing spectral features (for details, see Table 1 and Appendix S1: Section S1). These indices span pigment‐related (e.g., chlorophyll *a*/*b*, carotenoids), water‐related, and structural spectral features across the VIS, red‐edge, near‐infrared (NIR), and shortwave infrared (SWIR) ranges, thus capturing major axes of leaf functional variation. The indices were first calculated from the raw spectral data and subsequently adjusted using the same linear model as described above. Second, we used SWs, that is, treating each wavelength in the adjusted spectrum as a phenotype. Third, we used hierarchical spectral clustering with parallel analysis (HSC‐PA), a new data‐driven dimension reduction approach, developed from a method for the analysis of genetic associations with human facial features (Claes et al., 2018; Sero et al., 2019), which accounts for correlations within spectral data while retaining interpretable features. We applied HSC‐PA to the adjusted spectral data for downstream analyses.

**TABLE 1:** Six spectral indices used in index‐genome‐wide association studies (GWAS).

Index	Formula	Example of validated systems	References
NDWI	$\frac{R_{865}-R_{1614}}{R_{865}+R_{1614}}$	Crops, forests, wetlands, grasslands	Gao (1996), Zhang and Zhou (2019), Karthikeyan et al. (2020), Huete (2012), Adam et al. (2010), Gu et al. (2007), Helfenstein et al. (2022)
$CI_{\mathrm{re}}$	$\frac{R_{783}}{R_{704}}-1$	Crops, trees and vines, wetlands	Gitelson et al. (2001), Qian et al. (2021), Gitelson et al. (2005), Gitelson et al. (2003), DeLancey et al. (2019), Helfenstein et al. (2022)
CCI	$\frac{R_{560}-R_{664}}{R_{560}+R_{664}}$	Crops, trees, forests	Gamon et al. (2016), Dechant et al. (2020), Grabska et al. (2019), Helfenstein et al. (2022)
$\text{ARDSI}\_C_{\mathrm{ab}}$	$\frac{R_{750}-R_{730}}{R_{770}+R_{720}}$	Crops	Wan et al. (2021)
$\text{ARDSI}\_C_{w}$	$\frac{R_{1360}-R_{1080}}{R_{1560}+R_{1240}}$	Crops	Wan et al. (2021)
$\text{ARDSI}\_C_{m}$	$\frac{R_{2200}-R_{1640}}{R_{2240}+R_{1720}}$	Crops	Wan et al. (2021)

We then associated the resulting phenotypic data with 168,903 SNPs using multiple GWAS models based on the Genome Association and Prediction Integrated Tool (GAPIT) version 3 (Wang & Zhang, 2021). The GWAS models include general linear model (GLM, Price et al., 2006), mixed linear model (MLM, Yu et al., 2006), fixed and random model circulating probability unification (FarmCPU, Liu et al., 2016), and Bayesian information and linkage‐disequilibrium iteratively nested keyway (BLINK, Huang et al., 2019). Kinship matrices were automatically calculated by GAPIT using the Zhang method (Zhang et al., 2010) from the genotype data; these matrices were applied only in models that incorporate kinship (i.e., MLM). See more details of the four models in Appendix S1: Section S1.

To facilitate a direct and unbiased comparison of signal specificity between methods with vastly different phenotype numbers, we focused our detailed analysis on the top 50 unique SNPs identified by each approach. SNPs were ranked according to their minimum *p* value across all tested phenotypes. For these top 50 loci, we visualized and analyzed all associations that surpassed the significance level of the 50th ranked SNP (i.e., using the *p* value of the 50th SNP as a dynamic, method‐specific exploratory threshold).

For visualization and determination of statistical significance, multiple testing adjustments were conducted on 168,903 SNPs. This included a false discovery rate (FDR) adjustment (Benjamini & Hochberg, 1995) and the application of the PhenoSpD method (Nyholt, 2004; Zheng et al., 2018) to determine the significance threshold $\left(0.05/M_{\mathrm{eff}}\right)$, taking into account the effective number of independent trait calculations. The PhenoSpD method first derives the pairwise phenotypic correlation matrix from GWAS summary statistics using cross‐trait linkage disequilibrium (LD) score regression. Specifically, for two traits $t_{1}$ and $t_{2}$, the regression:

$$Z_{1j}Z_{2j}=a+b\times \text{LDscor}e_{j}+\epsilon_{j},$$

is calculated across SNPs j, where $Z_{1j}$ and $Z_{2j}$ are the *z* scores from GWAS summary statistics for each trait, and the intercept a provides an estimate of the phenotypic correlation between traits. This phenotypic correlation matrix is then subjected to spectral decomposition (SpD), in which the eigenvalues ($\lambda_{i}$) are used to calculate the effective number of independent tests:

$$M_{\mathrm{eff}}=\frac{{\left(\sum_{i=1}^{M}\lambda_{i}\right)}^{2}}{\sum_{i=1}^{M}{\lambda_{i}}^{2}},$$

where M is the total number of phenotypes. We used $0.05/M_{\mathrm{eff}}$ as the significance threshold.

SNPs with FDR‐adjusted *p* values below the significance threshold were considered significantly associated. For candidate gene selection, we extracted genes within a conservative ±100 kb of significant SNPs from the *N. attenuata* genome, consistent with the LD decay distance reported for the closely related *Nicotiana tabacum* (Lai et al., 2021). We then refined the candidate gene list using the *N. attenuata* Data Hub (http://nadh.ice.mpg.de/NaDH/) to filter out genes lacking transcript accumulation evidence in leaves under various conditions. Genes with relevant functions were then identified based on annotation information.

We also conducted GWAS using indices, and HSC‐PA segments with raw spectral data as a general comparison to assess the effect of removing known environmental effects (see Appendix S1: Section S1, Results from raw spectral data).

## RESULTS

### Phenotypic analysis

#### Variance partitioning and adjustment of raw spectra

**FIGURE 2:**
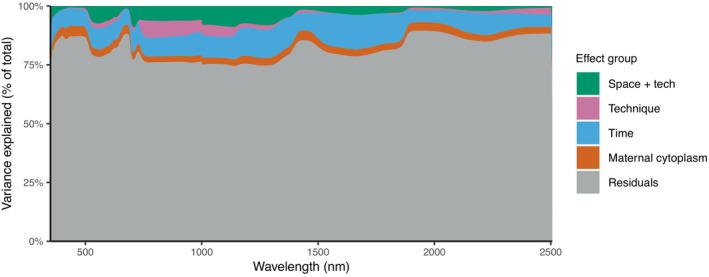
Variance partitioning across the spectral range of 350–2500 nm. Each column represents the cumulative percentage of variance for a given wavelength.

To advance our understanding of the phenotypic variation in leaf spectral traits and to remove the known environmental effects, we employed linear models. These models partitioned variance across the spectral range of 350–2500 nm, assessing contributions to cumulative multiple $R^{2}$ values (i.e., percent sum of squares) from measurement technique (LN), replicate and spatial effects and their interactions with measurement technique ($\mathrm{REP}+\mathrm{BA}+\mathrm{LN}:\left(\mathrm{REP}+\mathrm{BA}\right)$), measurement time (DT:CT), and maternal cytoplasm (MO) within the 616 plants. Figure 2 illustrates these components for each wavelength as stacked bars, which—given the large number of wavelengths—merge into a continuous stacked profile that collectively accounts for the total variance. Analysis of variance results indicated that residual phenotypic variance between plants, representing genetic and residual environmental contributions, accounted for 71.7%–89.5% of the total phenotypic variance.

#### Spectral characteristics of leaves

**FIGURE 3:**
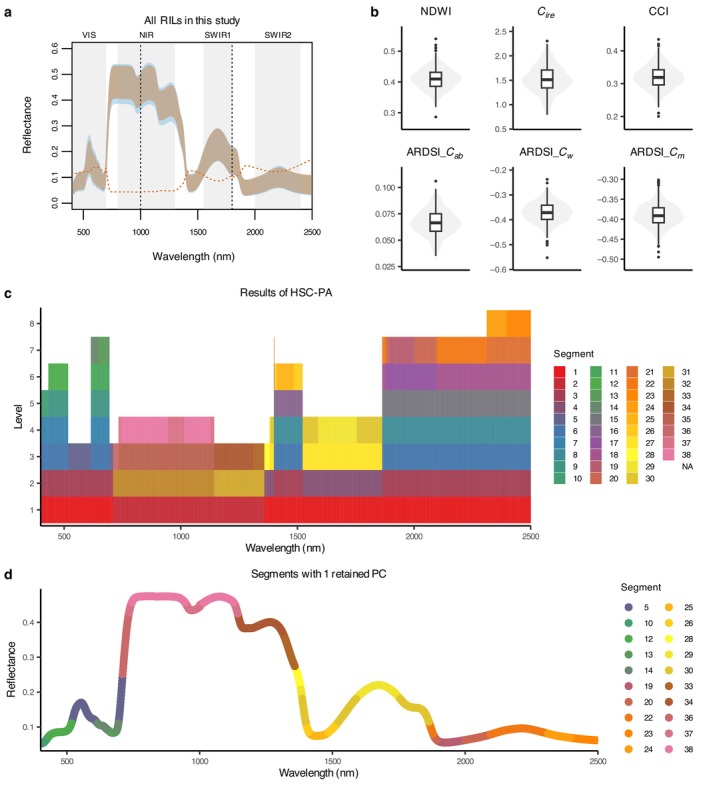
Spectra as phenotypes. (a) Leaf reflectance spectra for all 616 plants examined in this study. The orange shaded region illustrates the range of adjusted spectra, and the blue shaded region the range of raw spectra. The orange dotted line represents the coefficient of variation for the entire sample set. (b) Distribution of the six spectral indices used in the index‐genome‐wide association study (GWAS) approach. (c) Results from the hierarchical spectral clustering with parallel analysis (HSC‐PA). A total of 38 segments, differentiated by color, emerged from the clustering. Regions left blank (NA) indicate segments that retained one principal component (PC) in the prior level, halting further clustering. (d) Presentation of the 20 segments, shaped as example spectra, each maintaining one retained PC. Spanning the entire spectrum from 400 to 2500 nm, these segments served as phenotypes in the HSC‐PA‐GWAS approach to pinpoint associations with genetic variances. ARDSI, adaxial–abaxial reflectance difference spectral index; *C*_*ab*_, chlorophyll *a*/*b*; CCI, chlorophyll carotenoid index; CI_re_, chlorophyll index red‐edge; *C*_*m*_, dry matter; *C*_*w*_, water; NDWI, normalized difference water index; NIR, near‐infrared; RIL, recombinant inbred lines; SWIR, shortwave infrared; VIS, visible.

Our analyses focus on calculated leaf reflectance spectra spanning from 400 to 2500 nm, as the initial region (350–399 nm) has a poor signal‐to‐noise ratio and is commonly excluded from downstream analyses (Cavender‐Bares et al., 2016). This range includes the VIS, NIR, and SWIR regions. This broad spectral range captured variation among the 616 plants, shown in Figure 3a as an orange shaded region indicating the range of adjusted reflectance spectra for all samples. The blue shaded region indicates the range of raw reflectance spectra before adjustment with linear models. The NIR region showed more consistency in reflectance values among the plants, contrasting with greater variability in the VIS and SWIR regions.

The distribution of six selected spectral indices (Figure 3b), encapsulating attributes like water content and chlorophyll concentration, provided more specific information about leaf physiology. Note that the *y*‐axis represents the index values, which are specific to each index calculation and therefore cannot be directly compared across indices.

Our subsequent application of the HSC‐PA segmented the adjusted spectra into 38 distinct patterns. The blank (not applicable) areas are regions where further clustering was halted due to the retention of a singular principal component (PC) from a previous level. This approach allowed for a finer granularity in the spectral data representation while accounting for correlations among wavelengths. The hierarchical clustering process concluded at different levels, ranging from level 3 to level 8, indicating the varying depths of spectral similarities among the segments. Table 2 shows the detailed spectral region and potential associated features of the segments used in HSC‐PA‐GWAS. Appendix S1: Table S1 shows the spectral regions of all 38 segments.

**TABLE 2:** Spectral ranges of segments used in genome‐wide association studies (GWAS) with potential associated features.

Segment	Spectral range (nm)	Potentially associated physiological features^a^
5	518–614, 695–709	VIS–NIR: Chlorophyll *a*, chlorophyll *b*, carotenoids, anthocyanins; leaf structure
10	400–432	VIS: Chlorophyll *a*, chlorophyll *b*, carotenoids, anthocyanins
12	433–517	VIS: Chlorophyll *a*, chlorophyll *b*, carotenoids, anthocyanins
13	648–690	VIS: Chlorophyll *a*, chlorophyll *b*, carotenoids, anthocyanins
14	615–647, 691–694	VIS: Chlorophyll *a*, chlorophyll *b*, carotenoids, anthocyanins
19	1894–2000	SWIR: Water content
20	1880–1893, 2001–2096	SWIR: Nonpigment organic composition (e.g., lignin, cellulose, starch, protein, sugar, oil)
22	1400–1401, 1864–1879, 2097–2311	NIR–SWIR: Water content; leaf structure; nonpigment organic composition
23	2397–2500	SWIR: Water content
24	2312–2396	SWIR: Nonpigment organic composition
25	1412–1484	SWIR: Water content
26	1402–1411, 1485–1522	SWIR: Nonpigment organic composition
28	1359–1380	SWIR: Water content
29	1381–1385, 1590–1754	SWIR: Nonpigment organic composition
30	1386–1399, 1523–1589, 1755–1863	SWIR: Nonpigment organic composition
33	1311–1358	NIR: Water content
34	1144–1310	NIR: Leaf structure
36	710–733	NIR: Leaf structure
37	734–744, 946–1014, 1129–1143	NIR: Leaf structure
38	745–945, 1015–1128	NIR: Leaf structure

a The associated features are based on common findings (Jacquemoud & Ustin, 2019) and are intended to indicate physiological properties of leaves known to affect the given spectral ranges. These interpretations are subject to various factors and should not be considered definitive.

Abbreviations: NIR, near‐infrared; SWIR, shortwave infrared; VIS, visible.

By visualizing the 20 segments that each retained only a single PC, we were able to span the entire spectrum from 400 to 2500 nm. These segments, parsed into several interpretable regions, were then used for the HSC‐PA‐GWAS approach, offering phenotypic data for uncovering associations with underlying genetic variations. For comparison, the distributions of the six indices and the HSC‐PA results from raw spectral data (prior to environmental correction) are shown in Appendix S1: Figure S1.

#### Effective number of independent traits ($M_{\mathrm{eff}}$)

To account for multiple testing due to the analysis of multiple phenotypes in our GWAS approaches, we determined the effective number of independent traits, denoted as $M_{\mathrm{eff}}$, using the PhenoSpD method (Zheng et al., 2018). For the Index‐GWAS approach, which employed six distinct spectral indices, the effective number of independent tests, $M_{{\mathrm{eff}}_{\mathrm{ind}}}$, was determined to be 5.0. In the single‐wavelength GWAS (SW‐GWAS) approach, the effective number, $M_{{\mathrm{eff}}_{\mathrm{sw}}}$, was notably higher at 999.9. Finally, for the hierarchical spectral clustering with parallel analysis GWAS (HSC‐PA‐GWAS) approach, which clusters the spectra based on similarity, the effective number, $M_{{\mathrm{eff}}_{\mathrm{hsc}-\mathrm{pa}}}$, was found to be 10.5, reflecting the reduced dimensionality and the focus on major patterns of variation. These calculated $M_{\mathrm{eff}}$ values are used to set a threshold ($0.05/M_{\mathrm{eff}}$) for significant associations.

### SNP marker analysis

The kinship relationships among the 616 plants are visualized in Appendix S1: Figure S2. Through a heatmap, the genetic relatedness between individuals is presented. Most values in the histogram gravitate towards zero, indicating a consistent and minimal genetic difference between plants. This uniform distribution of kinship values affirms the thorough genetic mixing of the PLs in the MAGIC population and is consistent with the finding that this population does not display obvious structure (Ray et al., 2023).

Appendix S1: Figure S3 presents the frequency of heterozygosity of individual and markers, while Appendix S1: Figure S4 illustrates the LD decay over distance.

### Results of genome‐wide association studies

#### Index‐GWAS results

**FIGURE 4:**
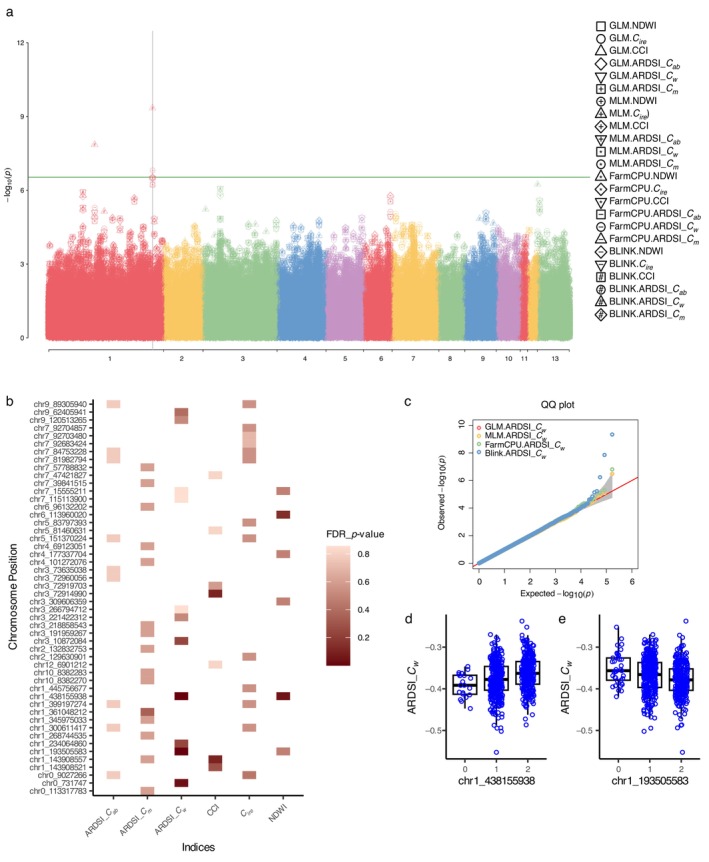
Index‐genome‐wide association study (GWAS) results. (a) Manhattan plot showing the $–\log_{10}\left(p_{\text{value}}\right)$ of single nucleotide polymorphism (SNP) associations for the six indices across various models. (b) Visualization of associations for the top 50 unique SNPs ranked by significance. Only associations surpassing the *p* value of the 50th ranked SNP ($5.7\times 10^{-5}$) are displayed. The *x*‐axis represents the six indices, the *y*‐axis the corresponding associated SNPs. The gradient red color scale represents the false discovery rate (FDR)‐adjusted *p* values. (c) Quantile–quantile (QQ) plot contrasting the observed versus the expected $–\log_{10}\left(p_{\text{value}}\right)$ for the GWAS analysis on the ${{\text{ARDSI}}_{C}}_{w}$ index. The diagonal line represents the expected distribution under the null hypothesis. The different color schemes depict the models used: red for general linear model (GLM), yellow for mixed linear model (MLM), green for fixed and random model circulating probability unification (FarmCPU), and blue for Bayesian information and linkage‐disequilibrium iteratively nested keyway (BLINK). Phenotypic distribution for two significant markers, chr1_438155938 (d) or chr1_193505583 (e), in association with ${\text{ARDSI}}_{C_{w}}.$ 0 represents a homozygous allele from the reference, 1 a heterozygous allele, and 2 a homozygous alternative allele. ARDSI, adaxial–abaxial reflectance difference spectral index; $C_{\mathrm{ab}}$, chlorophyll *a*/*b*; $C_{m}$, dry matterl; $C_{w}$, water; $CI_{\mathrm{re}}$, chlorophyll index red‐edge; CCI, chlorophyll carotenoid index; NDWI, normalized difference water index.

The Manhattan plot presented in Figure 4a shows the associations between the SNPs and the six indices (NDWI, CCI, $CI_{\mathrm{re}}$, $\text{ARDSI}\_C_{\mathrm{ab}}$, $\text{ARDSI}\_C_{w}$, and $\text{ARDSI}\_C_{m}$) using four models (GLM, MLM, FarmCPU, and BLINK). Several genomic regions displayed distinct associations according to the $-\log_{10}\;\left(p\;\text{values}\right)$.

In Figure 4b, we visualized the genetic associations for the top 50 unique SNPs ranked by significance across all indices. To facilitate a clear comparison, we employed a dynamic threshold based on the *p* value of the 50th ranked SNP ($p<5.7\times 10^{-5}$). In total, these top 50 unique SNPs corresponded to 61 associations surpassing the dynamic threshold. The gradient color scale indicates the association's intensity, with deeper hues indicating smaller FDR‐adjusted *p* values. This representation provides a concise overview of the genomic regions that are most strongly correlated with each spectral index.

Among these top associations, the index $\text{ARDSI}\_C_{w}$ displayed two associations lower than the significance threshold: 0.05/5.0=0.01, with two SNPs in chromosome 1. The first SNP was located at chr1_193505583, near Niat3g_08138, annotated as a Zeta toxin domain‐containing protein. The second was at chr1_438155938, close to Niat3g_17277 (TPR_REGION domain‐containing protein) and Niat3g_17282 (Exocyst subunit Exo70 family protein). Furthermore, $\text{ARDSI}\_C_{w}$ was chosen as a representative example for the quantile–quantile (QQ) plots in Figure 4c,d. This plot serves as a diagnostic tool for the GWAS results. Each model is represented by a unique color: red for GLM, yellow for MLM, green for FarmCPU, and blue for BLINK. The alignment of the observed points with the diagonal line indicates that the GWAS results are mostly consistent with the expectations under the null hypothesis. However, deviations can pinpoint regions with stronger genetic signals. Figure 4e,f shows the phenotypic distributions for two significant markers, where 0 represents a homozygous reference allele, 1 a heterozygous allele, and 2 a homozygous alternative allele.

#### SW‐GWAS and HSC‐PA‐GWAS results

**FIGURE 5:**
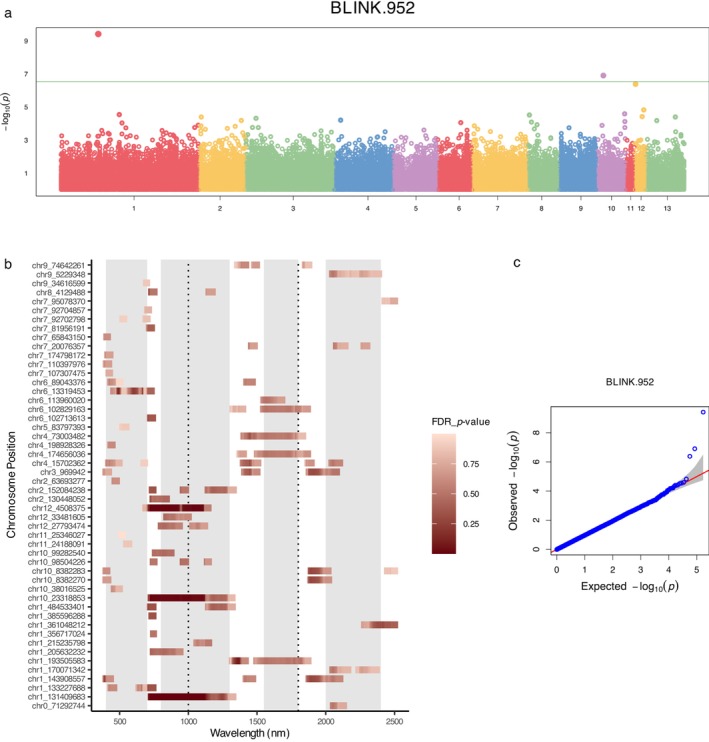
Single‐wavelength genome‐wide association study (SW‐GWAS) results. (a) Manhattan plot showing the $-\log_{10}\left(p\_\text{value}\right)$ of single nucleotide polymorphism (SNP) associations with Bayesian information and linkage‐disequilibrium iteratively nested keyway (BLINK) for wavelength 952 with the strongest association. (b) Visualization of associations for the top 50 unique SNPs ranked by significance. Only associations surpassing the *p* value of the 50th ranked SNP ($2.1\times 10^{-5}$) are displayed. The *x*‐axis represents the spectral wavelengths from 400 to 2500 nm, while the *y*‐axis displays the corresponding associated SNPs. The gradient red color scale represents the false discovery rate (FDR)‐adjusted *p* values. (c) Quantile–quantile (QQ) plot of the BLINK model contrasting the observed versus the expected $-\log_{10}\left(p\_\text{value}\right)$ for the GWAS analysis on the wavelength 952 nm. The diagonal line represents the expected distribution under the null hypothesis.

In the SW‐GWAS analysis, the BLINK model was selected to investigate the genetic correlations with 2101 spectral wavelengths spanning from 400 to 2500 nm. Figure 5a highlights the genetic associations for the spectral wavelength at 952 nm, which displayed the strongest association among all phenotypes, serving as an example.

Figure 5b offers an in‐depth depiction of associations for the top 50 unique SNPs ranked by statistical significance. To allow for a fair comparison, we applied a dynamic threshold corresponding to the *p* value of the 50th ranked SNP ($p<2.1\times 10^{-5}$). The *x*‐axis represents the spectral wavelengths, the *y*‐axis the associated SNPs, providing a holistic view of the spectral regions correlated with each SNP. These top 50 unique SNPs generated a total of 7800 significant associations. The majority of SNPs are linked to one or multiple continuous spectral regions, forming long horizontal stripes. This pattern visually confirms the massive statistical redundancy inherent in single‐wavelength GWAS, where a single genetic locus is associated with tens to hundreds of highly correlated wavelengths. Furthermore, correlations with the same SNP spanning more distant spectral regions may also reflect underlying biological processes and warrant further investigation. The significance level, adjusted by $M_{\mathrm{eff}}$, stands at $5.0\times 10^{-5}$. Notably, no SNP met this threshold. The diagnostic QQ plot for wavelength 952 nm is presented in Figure 5c.

**FIGURE 6:**
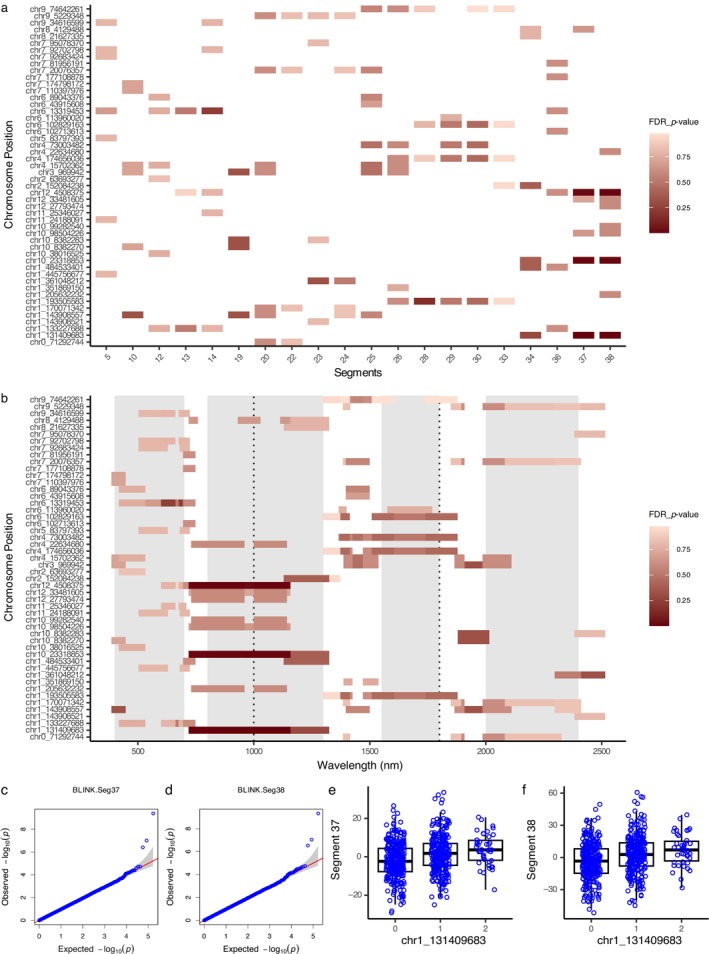
Hierarchical spectral clustering with parallel analysis genome‐wide association study (HSC‐PA‐GWAS) results. (a) Detailed visualization of associations for the top 50 unique single nucleotide polymorphisms (SNPs) ranked by significance. Only associations surpassing the *p* value of the 50th ranked SNP ($3.3\times 10^{-5}$) are displayed. The *x*‐axis enumerates the 20 segments, while the *y*‐axis displays the corresponding associated SNPs. The gradient red color scale represents the false discovery rate (FDR)‐adjusted *p* values. (b) An alternative representation of the associations from (a) with the *x*‐axis showcasing the spectral wavelengths, providing insights into the specific spectral regions of the associations. Quantile–quantile (QQ) plots highlighting the observed versus expected $-\log_{10}\left(p\_\text{value}\right)$ for GWAS analysis on Segment 37 (c) or Segment 38 (d). Phenotypic distribution for the significantly associated marker (chr1_131409683) in association with Segment 37 (e) or Segment 38 (f). 0 represents a homozygous allele from the reference, 1 a heterozygous allele, and 2 a homozygous alternative allele.

In the HSC‐PA‐GWAS, Figure 6a shows the associations of the top 50 unique SNPs ranked by significance, using its corresponding dynamic threshold ($p<3.3\times 10^{-5}$). In contrast to SW‐GWAS, these 50 SNPs corresponded to only 115 associations distributed over specific segments. Notably, 42 (84%) of the top 50 SNPs were identical between the SW‐GWAS and HSC‐PA‐GWAS results. Focusing on segment associations obscures spectral information, and so Figure 6b maps these associations back to their corresponding spectral wavelengths for better interpretation. This visualization facilitates the identification of specific spectral regions linked to each SNP. The significance level, after adjusting for $M_{\mathrm{eff}}$, is set at $4.7\times 10^{-3}$. Two associations, the SNP chr1_131409683 with Segments 37 and 38 (734–1143 nm), surpassed this threshold. The QQ plots for Segments 37 and 38, as depicted in Figure 6c,d, serve a dual purpose of validation and diagnostic assessment. Figure 6e,f shows the phenotypic distributions of the significant marker (chr1_131409683) affiliated with Segments 37 and 38. In this distribution, the numbers 0, 1, and 2 represent homozygous alleles as the reference, heterozygous alleles, and homozygous alternative alleles, respectively. The majority of the RILs are assigned reference or heterozygous alleles at this site, as evidenced by the greater number of points in classes 0 and 1. A clear difference in the mean values of these three classes is evident in the boxplot.

To further elucidate the genetic association indicated by HSC‐PA‐GWAS, we searched for potential candidate genes within a ±100‐kb window surrounding the SNP chr1_131409683. This led to the identification of one gene of interest: Niat3g_06527 (more annotation information can be found in Appendix S1: Table S2). This gene is annotated as carbonic anhydrase (CA).

We broadened our analysis to include the top 10 associations from HSC‐PA GWAS with the smallest FDR‐adjusted *p* values (see Appendix S1: Table S2). The top six associations are linked to Segment 37 or Segment 38 in the red‐edge and NIR regions. In addition to the significantly associated locus (chr1_131409683), these associations involve two SNPs: chr12_4508375 and chr10_23318853. Furthermore, the significantly associated locus (chr1_131409683) is also associated with Segment 34 (1144–1310 nm), which lies immediately after Segments 37 and 38 and remains within the NIR region.

## DISCUSSION

### HSC‐PA‐GWAS for discovering genetic associations with spectral variation

Here, we developed HSC‐PA‐GWAS as a new approach that retains the information in spectra in a biologically interpretable form without losing meaningful information, and without requiring a priori knowledge to target specific traits, while maintaining sufficient statistical power to identify potentially meaningful associations. Our comparative analysis of the top 50 unique SNPs identified by multiple both methods for spectral data treatment provides compelling evidence for the efficacy of HSC‐PA. First, the methods for spectral data treatment demonstrated high consistency, with 42 (84%) of the top 50 SNPs identified by HSC‐PA being identical to those found by SW‐GWAS. Second, the dynamic thresholds derived from the 50th ranked SNP were remarkably similar between SW‐GWAS ($p<2.1\times 10^{-5}$) and HSC‐PA‐GWAS ($p<3.3\times 10^{-5}$). This suggests that despite the significant reduction in dimensionality, HSC‐PA retains a level of detection sensitivity comparable to the single‐wavelength approach.

However, the HSC‐PA method surpasses SW‐GWAS in several key aspects. One of its strengths is its ability to reduce data dimensionality while accounting for phenotypic correlations within spectra. This advantage is clearly illustrated by the contrast in association patterns: while the top 50 SNPs in SW‐GWAS corresponded to over 7800 significant associations—manifesting as continuous, redundant spectral “stripes” (Figure 5b)—HSC‐PA condensed these signals into approximately 115 discrete, interpretable segment associations (Figure 6a). By transforming multiple correlated wavelengths into a single value, typically represented by the first PC, the HSC‐PA method mitigates the challenges of multiple testing and correlated phenotypic data and thus increases statistical power. By aggregating information across multiple wavelengths, the HSC‐PA method offers a more comprehensive and potentially more accurate representation of the underlying traits with its ability to discover the correlation structure among wavelengths from data. The resulting genetic associations encompass a broader portion of the spectrum than those derived from indices, which focus on specific wavelength combinations, or from SW, which do not behave independently.

This methodological advance addresses a critical challenge in the field. Understanding the genetic foundation of leaf spectral diversity will facilitate advances in biodiversity monitoring and phenotyping, but determining appropriate approaches to extract genetic information from complex, context‐dependent spectral phenotypes is a critical step (Li, Czyz, et al., 2023; Li, Czyż, et al., 2023). SW‐GWAS, while straightforward, is limited by the high dimensionality of spectral data and does not match the spectral resolution of instruments, typically ranging from 3 to 10 nm. Subsampling can simplify data and enhance signal‐to‐noise ratios, yet risks omitting key data points and does not account for correlation within spectra; and the choice of bands for subsampling and the resulting number of traits require consideration and testing. It is common to subsample the continuous spectral data to 1‐nm intervals, as done here for the SW‐GWAS and as a basis for other analyses, because 1 nm is the subsampling interval provided in the calibrated output data from the ASD FieldSpec and other field spectrometers. Cavender‐Bares et al. (2016) subsampled leaf spectra at a larger interval of 5 nm prior to PLS‐DA and evolutionary analysis and stated that the decision regarding the interval width (1, 2, 5, or 10 nm) did not significantly impact the results. Czyż et al. (2020) used a coarser subsampling interval of 10 nm to estimate the predictive power of spectral bands for genetic structure, based on the minimum spectral resolution of the instrument. Spectral indices provide a guided means to subsample spectra, selecting targeted wavelengths to derive ratios that can be related to specific traits, but these target a small portion of the spectrum and require validation if they are to be directly interpreted in terms of the targeted traits. Untargeted dimensionality reduction methods like PCA yield complex components challenging for biological interpretation (Li, Czyz, et al., 2023, Li, Czyż, et al., 2023), and varimax rotation is of limited value given the complexity of spectral wavelength loading on the components. Partial least squares regression (PLSR) is another alternative, often combined with discriminant analysis or beta regression models, but it requires orthogonal trait measurements for the regression and carries the risk of overfitting (Cavender‐Bares et al., 2016; Stasinski et al., 2021; Wang et al., 2020).

Comparing the HSC‐PA method with established methods, such as an approach combining PLSR and linear regression to interpret spectral features in terms of other traits (Verrelst et al., 2019), indicates that HSC‐PA has an advantage in at least two ways: supporting human interpretation of data‐derived spectral features and thus genetic associations, and allowing for experimental designs aimed at discovering associations with novel trait variation or where there are limitations on producing appropriate validation datasets required for regression. Verrelst et al. (2019) also emphasized the importance of nonparametric regression and machine learning methods, like the Kernel ridge regression (KRR) and Gaussian process regression (GPR), for their ability to capture nonlinear relationships in spectral data. These methods, while powerful, often require careful tuning, can be computationally intensive, and carry the danger of overfitting. The HSC‐PA method offers a data‐driven yet human‐interpretable approach with the caveat that its criterion for simplification is linear (dimensionality in a PCA). As discussed in studies on human facial shapes, from which the HSC‐PA method was adapted (Claes et al., 2018), this methodological shift allows for a transition from a global to a local understanding of spectral variation, and a holistic view of its genetic basis.

### Spectra adjustment and genetic associations

In this study, we used replicated plants of a single tester genotypes of *N. attenuata* as “phytometers” to account for small‐scale (2 × 2 planting positions) spatial variation across an experimental field. In addition, we accounted for differences in other environmental variables such as measurement time as well as for maternal effects. By applying a linear model across the full spectral range and using the residuals as adjusted spectra for downstream analyses, we were able to reduce the influence of confounding environmental variables. This correction, which would have been difficult to incorporate directly into a mixed‐model GWAS analysis, substantially altered many results, including derived phenotypes and GWAS associations, leading to different segment definitions and candidate genes in comparison with directly conducting GWAS on spectral data without prior correction for nongenetic effects (Appendix S1: Section S1, Results from raw spectral data). Such shifts underscore the importance of accounting for nongenetic effects in spectral–genomic studies, as failing to do so can lead to markedly different—and potentially misleading—biological conclusions. Here, we were able to do so both by using a phytometer and by accounting for environmental variables dictated by the experimental and measurement design; while a genetically invariable phytometer is rarely an option in ecological studies, it should usually be feasible to account for quantitative environmental and experimental effects on phenotype values.

Advanced genetic association models considering epistasis, akin to HSC‐PA's handling of spectral correlations, could enhance GWAS power, sensitivity, and interpretability, with advances in machine learning opening new possibilities (Libbrecht & Noble, 2015; Wu et al., 2021). Random forest models have already been used for analyzing complex genetic effects on multivariate phenotypes, though their significance interpretation differs (Brieuc et al., 2018; Wang et al., 2013). The “Epi‐MEIF” approach by Saha et al. (2022), using conditional inference forests for epistasis in complex phenotypes, offers a method for significant association identification, accommodating multiple loci testing as an add‐on to conventional single‐locus models like those in GAPIT.

### Ecological significance of findings in *N. attenuata*

We discovered a significant association between the SNP chr1_131409683 and Segments 37 and 38 (734–1143 nm) in the HSC‐PA‐GWAS. One interesting candidate gene was identified within 100 kb of this SNP: Niat3g_06527 is annotated as a CA. CAs catalyze the reversible hydration of CO₂ to bicarbonate and protons (CO₂ + H₂O ⇌ HCO₃^−^ + H^+^) (Supuran, 2004). In plants, multiple CA isoforms are found in the chloroplast stroma, thylakoid membranes, cytosol, and other compartments (Fabre et al., 2007; Rudenko et al., 2015). Chloroplastic CAs are thought to facilitate the rapid supply of CO₂ to Rubisco by accelerating the interconversion between dissolved inorganic carbon species, thereby supporting the Calvin–Benson cycle (Badger & Price, 1994). In C₄ and CAM plants, CA activity in mesophyll cells is also critical for providing bicarbonate to phosphoenolpyruvate carboxylase during the initial fixation step (Hatch & Burnell, 1990). In C₃ species such as *N. attenuata*, stromal and thylakoid‐associated CAs have additionally been implicated in maintaining photosynthetic efficiency under fluctuating light and CO₂ conditions, and in the regulation of pH and ion balance in the thylakoid lumen (Tiwari et al., 2016).

The associated spectral range, 734–1143 nm, spans the red‐edge and NIR regions, which are sensitive to changes in leaf internal structure and chlorophyll content. Genetic variation in a chloroplast‐localized CA could influence CO₂ assimilation rates and downstream effects on chlorophyll concentration, mesophyll cell structure, or water status, any of which can alter reflectance in this part of the spectrum. The link between Niat3g_06527 and these spectral regions therefore suggests that natural allelic differences in CA function may contribute to variation in photosynthetic performance and leaf optical properties in *N. attenuata*.

In the context of the ecology of *N. attenuata*, understanding the genetic basis of spectral variation can provide insights into the adaptive mechanisms of this wild plant. The significant association of a SNP close to a gene annotated as CA, with a spectral range spanning the red‐edge and NIR regions—wavelengths closely linked to chlorophyll content, canopy structure, and photosynthetic capacity—may reflect evolutionary pressures that have shaped the species' genetic architecture to optimize carbon fixation and water‐use efficiency, enhancing its survival in the variable and often harsh environments of its native habitat. Our extended analysis of the top 10 SNPs has unveiled additional genetic associations that may play important roles in the ecological adaptation and survival of *N. attenuata*.

Two other statistically significant associations were discovered with the $\text{ARDSI}\_C_{w}$ index, an index of water content that uses the wavelengths 1080, 1240, 1360, and 1560 nm, which are mostly (except for 1080 nm) in the range of wavelengths longer, that is, further into the infrared region, than those comprising Segments 37 and 38. The two associated SNPs, both also on chromosome 1, were located in the vicinity of the gene Niat3g_08138 (chr1_193505583), annotated as a zeta toxin domain‐containing protein, and the genes Niat3g_17277 annotated as TPR_REGION domain‐containing protein and Niat3g_17282 annotated as Exocyst subunit Exo70 family protein (chr1_438155938). Proteins with these domains are documented to serve a wide variety of functions in defense, growth and development, and cellular function.

It is interesting that the strongest associations were all identified between loci on chromosome 1 and wavelengths longer than those of VIS light, in the red‐edge to infrared portions of the leaf spectrum, that are influenced by micromorphology, structure, abundant nonpigment organic constituents, and water content. The strongest associations were within regions of high signal:noise ratio in spectral reflectance, that is, where the detection of phenotypic differences should be most robust. This alignment suggests that the detectability of genetic effects on leaf spectra is tightly coupled to measurement precision, and serves as an internal validation that the identified associations represent genuine biological signals rather than measurement artifacts.

### Limitations and outlook

One limitation of our study lies in the phenotypic interpretation for biological meanings specific to the samples we used. The six spectral indices we selected are derived from existing literature and have not been validated in *N. attenuata* generally, or for this dataset. Without chemical or other analyses to specifically quantify the constituents represented in the indices, such as chlorophyll, our interpretations remain speculative. Establishing causal links between candidate genes and specific biological processes is inherently difficult, particularly in ecological systems where multiple interacting factors may influence spectral traits (Hoban et al., 2016). Moreover, while the HSC‐PA‐GWAS approach has identified potential candidate genes, any influence of these genes on the traits of interest remains to be verified, for example, through the generation and testing of knockout, knockdown, and overexpression lines. The associations identified via our exploratory rank‐based comparison serve as hypotheses for functional validation rather than definitive causal links.

While GWAS with a MAGIC population does not require replication of genotypes, the absence of such replicates prevented us from testing variation between genotypes directly using the analysis of variance approach. Nevertheless, by removing small‐scale environmental variation using replicated phytometers of a single genotype as well as other environmental variation and maternal effects, the residual phenotypic differences between plants were likely due to their genetic differences rather than further environmental causes. However, we acknowledge that leaf spectral traits likely possess a highly polygenic architecture resulting from the integration of multiple biochemical and morphological pathways; the MAGIC design provides high power to detect individual genetic associations, but likely disrupts the architecture of polygenic traits found in the parent genotypes. Our field experimental design prioritized the removal of environmental variance to identify robust, constitutive signals. However, this approach (as the one incorporating environmental variance directly in mixed models during GWAS) does not remove any genotype‐by‐environment (G×E) interactions that may limit the power to detect minor polygenic effects compared to controlled laboratory studies with lower environmental variance and therefore also smaller genotype‐by‐environment interactions.

We identified unexpectedly high heterozygosity levels (mean around 0.5, see Appendix S1: Figure S3) in the dataset, potentially due to the low‐pass sequencing (mean 0.5× coverage) of the RILs (Ray et al., 2023). The significant data gaps (about 95% missing data) necessitated imputation from PLs, which were not highly inbred, likely inflating heterozygosity estimates in the RILs. While the imputed genotype matrix was accurate for known quantitative trait loci (Ray et al., 2023), the sequencing and imputation approach introduces uncertainty in true heterozygosity levels. Similarly, this sparse genomic coverage limits the resolution of long‐range LD, likely resulting in an underestimation of LD decay distances in our dataset (Appendix S1: Figure S4). Consequently, we adopted a conservative candidate gene search window (±100 kb) based on literature from related species rather than relying strictly on internal LD estimates. Factors like residual heterozygosity and heterozygote hotspots, seen in species like maize (Liu et al., 2018), may also influence these high heterozygosity readings in the *N. attenuata* MAGIC RIL population. Future studies with higher sequencing depth could more accurately determine homozygosity and heterozygosity in this population, aiding in dissecting genetic architecture and validating GWAS findings.

Looking forward, there is great potential for deepening our understanding of leaf spectral data using the *N. attenuata* system. Our analysis indicates relatively large variance in blue light reflectance and perhaps related photosynthetic traits among the wild genotypes contributing to the *N. attenuata* MAGIC population, as discussed previously (Li, Czyz, et al., 2023; Li, Czyż, et al., 2023). Variation in conserved and essential traits, which can be mediated by variants of regulatory genes embedded in coexpression networks (Ray et al., 2023), may lend different genotypes a competitive advantage under changing environmental conditions. The significant associations identified here thus indicate genotype and phenotype targets for novel and interesting in‐depth functional studies that could help us to better understand plant survival strategies. A combination of natural variants, genetic modification, and laboratory assays, such as gene expression analyses (Schena et al., 1995), protein–protein interactions (Fields & Song, 1989), and chromatin immunoprecipitation assays (Solomon et al., 1988), comprises the gold standard for functional testing and validation of discovered genetic associations. At this stage, the associations reported here represent hypotheses that should be subjected to these tests.

Unraveling the genetic basis of leaf spectral properties has far‐reaching implications and could, for example, revolutionize plant biodiversity monitoring, ecophysiology, and adaptation research, and help to identify genotypes resilient to stresses associated with global change. Our study sets the stage for future research aimed at deciphering these links by presenting a widely applicable new method for data‐driven discovery of associations which can be integrated with existing and novel tools to discover genetic associations and support moving from association to prediction or causation.

## CONCLUSIONS

In our study, we explored the genetic basis of leaf spectral properties in *N. attenuata* by associating spectral phenotypes with SNP genotypes in GWAS during a field experiment. We analyzed leaf reflectance spectra from 400 to 2500 nm, identifying regions correlating with leaf composition and function. We developed the hierarchical spectral clustering with parallel analysis (HSC‐PA) method to manage the data's high dimensionality and correlation, proving more effective than other methods like spectral indices or individual wavelengths. Our results demonstrate that while single‐wavelength approaches suffer from massive statistical redundancy (where single loci associate with hundreds of wavelengths), HSC‐PA effectively condenses these signals into discrete, interpretable features, offering greater specificity and statistical power.

Our findings included a significant SNP association in the red‐edge to NIR spectral range (734–1143 nm), a region sensitive to chlorophyll content, canopy structure, and photosynthetic efficiency. The most significant SNP lies near the Niat3g_06527 gene, annotated as CA, an enzyme central to photosynthetic carbon fixation and stomatal regulation. While leaf spectral traits are likely highly polygenic and influenced by environmental plasticity, our ability to identify a biologically plausible major locus in a field setting highlights the potential of the HSC‐PA approach. These results require further validation, but they raise the intriguing possibility that variation in red‐edge/NIR reflectance could be linked to differences in carbon assimilation and water‐use strategies in *N. attenuata*, potentially reflecting adaptations to its native, resource‐variable environments.

This study introduces a method and framework that can be broadly applied to attain deeper understanding of the genetic underpinnings of leaf spectral properties. The associations identified here using an advanced genetic toolset built for a native plant, and studied in a field experiment within the plant's natural range, have the potential to advance our understanding of the genetic mechanisms underlying photosynthetic efficacy and related growth and defense strategies. By providing empirical evidence linking specific genetic variants to spectral phenotypes in a wild plant, our work validates the fundamental premise of “spectranomics”—that functional plant traits are spectrally detectable and genetically determined—and can support the development of remote sensing as a scalable proxy for monitoring functional and genetic biodiversity.

## AUTHOR CONTRIBUTIONS

*Conceptualization*: Ian T. Baldwin, Meredith C. Schuman, Michael E. Schaepman. *Data curation*: Bernhard Schmid, Cheng Li, Rishav Ray. *Formal analysis*: Bernhard Schmid, Cheng Li. *Funding acquisition*: Ian T. Baldwin, Michael E. Schaepman. *Investigation*: Ewa A. Czyż, Meredith C. Schuman. *Methodology*: Bernhard Schmid, Cheng Li, Ewa A. Czyż, Meredith C. Schuman. *Project administration*: Meredith C. Schuman. *Resources*: Ian T. Baldwin, Michael E. Schaepman, Rayko Halitschke. *Supervision*: Meredith C. Schuman. *Visualization*: Cheng Li. *Writing—original draft*: Cheng Li. *Writing—review and editing*: Bernhard Schmid, Cheng Li, Ewa A. Czyż, Ian T. Baldwin, Meredith C. Schuman, Michael E. Schaepman, Rayko Halitschke, Rishav Ray.

## CONFLICT OF INTEREST STATEMENT

The authors declare no conflicts of interest.

## Supporting information


Appendix S1.

## Data Availability

Processed spectral data, genotypes data, and code (Li, 2026a) are archived in Zenodo at https://doi.org/10.5281/zenodo.18612876. Spectral measurement data underlying the results presented in this article (Li, 2026b) are available in Zenodo at https://doi.org/10.5281/zenodo.18596421. The plant lines used in this study are maintained at the Max Planck Institute for Chemical Ecology and because seed stocks are limited, they are not publicly archived but may be available upon reasonable request, subject to availability and institutional approval; requests should be directed to the institute's greenhouse facility (currently Danny Kessler; email: dkessler@ice.mpg.de) and should explicitly reference the *Nicotiana* MAGIC lines.
